# A novel constrained genetic algorithm-based Boolean network inference method from steady-state gene expression data

**DOI:** 10.1093/bioinformatics/btab295

**Published:** 2021-07-12

**Authors:** Hung-Cuong Trinh, Yung-Keun Kwon

**Affiliations:** Faculty of Information Technology, Ton Duc Thang University, Ho Chi Minh 758307, Vietnam; Department of IT Convergence, University of Ulsan, Ulsan 680-749, Korea

## Abstract

**Motivation:**

It is a challenging problem in systems biology to infer both the network structure and dynamics of a gene regulatory network from steady-state gene expression data. Some methods based on Boolean or differential equation models have been proposed but they were not efficient in inference of large-scale networks. Therefore, it is necessary to develop a method to infer the network structure and dynamics accurately on large-scale networks using steady-state expression.

**Results:**

In this study, we propose a novel constrained genetic algorithm-based Boolean network inference (CGA-BNI) method where a Boolean canalyzing update rule scheme was employed to capture coarse-grained dynamics. Given steady-state gene expression data as an input, CGA-BNI identifies a set of path consistency-based constraints by comparing the gene expression level between the wild-type and the mutant experiments. It then searches Boolean networks which satisfy the constraints and induce attractors most similar to steady-state expressions. We devised a heuristic mutation operation for faster convergence and implemented a parallel evaluation routine for execution time reduction. Through extensive simulations on the artificial and the real gene expression datasets, CGA-BNI showed better performance than four other existing methods in terms of both structural and dynamics prediction accuracies. Taken together, CGA-BNI is a promising tool to predict both the structure and the dynamics of a gene regulatory network when a highest accuracy is needed at the cost of sacrificing the execution time.

**Availability and implementation:**

Source code and data are freely available at https://github.com/csclab/CGA-BNI.

**Supplementary information:**

Supplementary data are available at *Bioinformatics* online.

## 1 Introduction

Recent high-throughput sequencing technologies have yielded a mass of gene expression data (Hughes *et al.*, 2000; Quackenbush, 2001), which provides an opportunity to investigate the underlying gene-regulatory mechanism from a system-level perspective. It is a challenging problem in systems biology to infer gene regulatory networks (GRNs) from these high-throughput gene expression data, and many computational methods have been developed for it (Banf and Rhee, 2017; Chai *et al.*, 2014; De Smet and Marchal, 2010). Specifically, the problem aims to infer not only a set of regulatory genes for a target gene (i.e. network structure inference), but also the regulatory rules between genes (i.e. network dynamics inference). In fact, most previous methods based on Bayesian networks (de Matos Simoes and Emmert-Streib, 2012; Liu *et al.*, 2016), graphical Gaussian models (Krämer *et al.*, 2009; Menéndez *et al.*, 2010), information theory approaches (Margolin *et al.*, 2006; Xiao *et al.*, 2016), correlation approaches (Yu *et al.*, 2017; Zuo *et al.*, 2014) and supervised learning approaches (Huynh-Thu *et al.*, 2010; Kotera *et al.*, 2012) have focused on only the network structure inference. On the other hand, a few methods using Boolean models (Kauffman, 1969; 1993), or differential equation-based models (Coddington and Levinson, 1955) have been proposed to predict both the network structure and dynamics. Although the differential equation-based models can offer higher precision predictions, they involve a large number of parameters which are difficult to optimize. In contrast, a Boolean model is the simplest model using the smallest number of parameters to simulate the dynamics of a system, and hence many Boolean model-based algorithms have been developed to infer the GRNs (Barman and Kwon, 2017; Berestovsky and Nakhleh, 2013; Han *et al.*, 2015). However, we note that most existing methods for Boolean network inference use time-series gene expression data as an input whereas a few studies proposed a Boolean network inference method using steady-state expression data (Almudevar *et al.*, 2011; Chevalier *et al.*, 2019; Lim *et al.*, 2016). A recent study (Chevalier *et al.*, 2019) addressed the synthesis of the Boolean networks from constraints on their domain and emerging dynamical properties of the resulting network, but it requires a prior knowledge about a network structure as well as the biological dynamical constraints, which depend on the assumptions about the phenotypes. Another study (Almudevar *et al.*, 2011) proposed a Bayesian method to infer a Boolean GRN from steady-state expression data by directly evaluating a model uncertainty. However, it was applicable to only small-scale networks consisting of a few genes because of the expensive computational cost in the Bayesian model. A swarming hill climbing search-based method (Lim *et al.*, 2016) was suggested to refine or reconstruct Boolean models from single-cell expression data. IT is basically a local search which iteratively improves a known network structure. In addition, it explores all neighborhood solutions of a currently selected Boolean model which limits the inference of large-scale networks. Taken together, the previous methods of inferring Boolean network from steady state expression have limitations in handling large-scale networks or in obtaining a priori biological information.

To overcome the limitation, we propose a novel constrained genetic algorithm-based Boolean network inference (CGA-BNI) method. Given steady-state gene expression data as an input, the method first identifies a set of path consistency-based constraints by comparing the gene expression level between the wild-type and the mutant experiments. Next, a genetic algorithm searches not only a network structure to meet the consistency-based constraints but also a set of Boolean update functions to best fit the gene expression dynamics. Our method is a global-search algorithm and intrinsically slow, thus we devised a heuristic mutation operation in the genetic algorithm for faster convergence and implemented the evaluation step in parallel by using Java multi-core programming to make CGA-BNI be applicable to larger networks. Through extensive simulations on three kinds of datasets such as the DREAM challenge data, the artificial data and the large-scale *E*. *coli* gene expression data, CGA-BNI showed consistently better performance than five other well-known existing methods, CellNOptR, ARACNE, GENIE3, BC3NET and BTR in terms of both structural and dynamics prediction accuracy. Considering that the execution time of CGA-BNI was second slowest among the five models, it is most suitable when a highest prediction accuracy is expected at the cost of sacrificing the execution time.

## 2 Materials and methods

In this section, we explain the basic concept of a Boolean network model employed in CGI-BNI and introduce the Boolean network inference problem from steady-state gene expression data.

### 2.1 A Boolean network model

A Boolean network is one of the simplest computational models to describe network dynamics (Kauffman, 1969; 1993), and it has been frequently used to investigate the complex behaviors of biological networks (Helikar *et al.*, 2008; Le and Kwon, 2011; Trinh *et al.*, 2014). It is represented by a directed graph $G=\left(V,A\right)$ where $V=\left\{v_{1},\;v_{2},\;\ldots ,\;v_{N}\right\}$ is a set of nodes and A is a set of ordered pairs of the nodes called directed links ($\left|V\right|$ and $\left|A\right|$ denote the number of nodes and links, respectively). A directed link $\left(v_{i},v_{j}\right)\in A$ represents a positive (activating) or a negative (inhibiting) regulation from $v_{i}$ to $v_{j}$. Each $v_{i}\in V$ has a state value of 1 (on) or 0 (off). The state of $v_{i}$ at time t+1 denoted by $v_{i}(t+1)$ is established by the values of $k_{i}$ other nodes $v_{i_{1}},\;v_{i_{2}},\;\ldots ,\;v_{i_{k_{i}}}$ with a link to $v_{i}$ at time t by a Boolean function $f_{i}:{\left\{0,1\right\}}^{k_{i}}\to \left\{0,1\right\}$ and the states of all nodes are synchronously updated. Here, we employed a nested canalyzing function (NCF) model (Kauffman *et al.*, 2004) to represent an update rule as follows:

fivi1t,vi2t,…,vikit=O1 if vi1(t)=I1 O2 if vi1(t)≠I1 and vi2(t)=I2 O3 if vi1t≠I1 and vi2t≠I2 and vi3t=I3 ⋮Oki if vi1t≠I1 and ⋯and viki-1t≠Iki-1 and vikit=IkiOdef otherwise 

where all $I_{m}$ and $O_{m}\;(m=1,2,\ldots ,k_{i})$ denote the canalyzing and canalyzed Boolean values, respectively, and $O_{\mathrm{def}}$ is set to $1-O_{k_{i}}$ in general. For convenience, we denote $f_{i}$ as $(I_{1},\;O_{1})(I_{2},\;O_{2})\cdots (I_{k_{i}},\;O_{k_{i}})O_{\mathrm{def}}$, which is a sequence of pairs of canalyzing and canalyzed values, followed by the default value. In this study, each NCF is randomized by specifying every $I_{m}$ and $O_{m}$ between 0 and 1 uniformly at random. We note that many molecular interactions were successfully represented by NCFs in previous studies (Samal and Jain, 2008; Trinh and Kwon, 2016).

A *network state* at time t can be denoted by an ordered list of state values of all nodes, $v\left(t\right)=\;v_{1}(t)v_{2}(t)\cdots v_{N}(t)\in {\left\{0,1\right\}}^{N}$. Since a synchronous update scheme was considered in this study, every network state transits to another network state through a set of Boolean update functions $F=\left\{f_{1},f_{2},\ldots ,f_{N}\right\}$ in a deterministic way. Hence, a network state trajectory starting from an initial network state eventually converges to either a fixed-point or a limit-cycle attractor. These attractors can represent diverse biological network behaviors such as multi-stability, homeostasis and oscillation. We define the attractor more rigorously as follows.

***Definition.*** Given a Boolean network$\;G=\left(V,A\right)$ where $V=\left\{v_{1},\;v_{2},\;\ldots ,\;v_{N}\right\}$ and A⊆V×V and a set of Boolean update functions $F=\left\{f_{1},f_{2},\ldots ,f_{N}\right\}$, let $v\left(0\right),\;v\left(1\right),\;\cdots ,\;$be a network state trajectory starting at $v\left(0\right)$. The *attractor* is defined as an ordered list of network states $\alpha =\left[v\left(\tau \right),v\left(\tau +1\right),\;\ldots ,v\left(\tau +p-1\right)\right]$ where $\tau =\underset{t}{\mathrm{minarg}(}v\left(t\right)=\;v\left(t+p\right))$ for some p with $v\left(i\right)\neq v\left(j\right)\;\mathrm{for}\;\forall i\neq j\in \left\{\tau ,\tau +1,\ldots ,\tau +p-1\right\}$ (herein, p is called a length of the attractor).

### 2.2 Boolean network inference problem

**Fig. 1.**
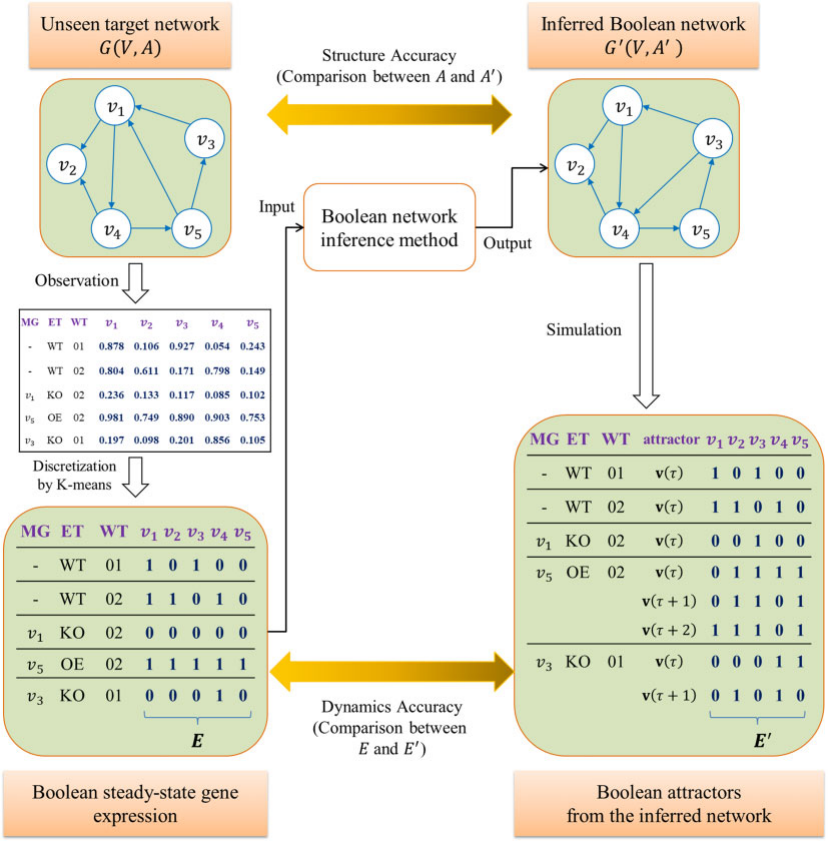
Overview of a Boolean network inference problem. An unseen target network $G\left(V,A\right)$ produces a steady-state gene expression dataset that is converted to a Boolean steady-state dataset E by a discretization method. An inference algorithm trains the Boolean dataset E along with experimental information as an input and infers a Boolean network $G^{'}\left(V,A^{'}\right)$ as an output. The inference performance is evaluated in terms of a structural accuracy by comparing the inferred connections $A^{'}$ to the true connections A and a dynamics accuracy by comparing the derived Boolean attractors $E^{'}$ to the observed data E. MG, ET, WT, KO and OE denote ‘mutated gene’, ‘experiment type’, ‘wild-type’, ‘gene knockout’ and ‘gene overexpression’, respectively

The Boolean network inference problem tackled in this study is a problem of inferring a Boolean network which best fits the observed steady-state gene expression data (Fig. 1). As shown in the figure, a steady-state expression dataset of real values can be obtained from an unseen underlying target network $G\left(V,A\right)$. The dataset is represented by a matrix consisting of N columns of genes and R rows of experiments. Then, it is transformed into a dataset of binary values by a discretization method and we herein used the K-means clustering-based discretization method (MacQueen, 1967), which groups all of the expression values of a gene into two clusters, and assigns 1 (the ‘on’ state) and 0 (the ‘off’ state) to the clusters having relatively higher and lower average values, respectively. As a result, we can construct the set of steady-state Boolean gene expression matrix E where a row corresponding to an experiment is represented by an N-dimensional Boolean expression vector i.e. $e\in {\left\{0,1\right\}}^{N}$. In addition, the experimental information about the mutated gene (available in case of a perturbation experiment), the type of experiments (i.e. one of wild-type or perturbation experiments) and the base wild-type experiment are annotated by ‘MG’, ‘ET’ and ‘WT’ fields, respectively. For example, the fifth experiment in Figure 1 describes a knockout perturbation subject to gene $v_{3}$ and the base wild-type experiment is ‘WT01’ which means the first wild-type experiment. A Boolean network inference method uses both the dataset E and the experimental information as inputs, and produces an inferred network $G^{'}\left(V,A^{'}\right)$ as a result. Unlike most previous methods which do not focus on the inference of a regulatory function, we defined the problem such that the inference result should include an estimated update Boolean rule for each gene because it is required to evaluate the dynamics accuracy of the inferred network. Specifically, the dynamics accuracy is calculated by comparing the Boolean steady-state expression in E and the attractor which is derived from the inferred network. Consider an arbitrary Boolean expression e∈E and let $e_{b}$ the Boolean expression of the base experiment of e (Note that $e_{b}$ is equivalent to e if e is the case of the wild-type experiment). To compute the corresponding attractor of the inferred network, the initial state of $G^{'}$ is specified to $e_{b}$ and the trajectory is simulated by the inferred update function until an attractor is obtained. For example, the third experiment in Figure 1 describes a knock-out perturbation (KO) experiment subject to $v_{1}$ where the base wild-type experiment is the second experiment denoted by WT02. Thus, the inferred network state is initialized to $e_{b}=\left[11010\right]$ by referring to the Boolean steady-state expression of WT02 in E. Then the gene $v_{1}$ is assumed to be knocked out and the attractor which $G^{'}$ converges to is examined. Assume that a fixed-point attractor $\left[00100\right]$ is found as shown in Figure 1. Then, it is compared with the Boolean expression of the third experiment in E, $e=\left[00000\right]$, to compute the dynamics accuracy. In this way, we obtain the attractor by simulating $G^{'}$ for each steady-state expression e∈E and construct the resultant set of attractors E'.

In this study, we consider two types of inference performance. The first one is the structural performance by comparing A and $A^{'}$, and we employed three well-known metrics, precision, recall and structural accuracy. Precision is defined as the ratio of correctly inferred connections out of all positive predictions, as follows:

(1) $$\mathrm{Precision}=\frac{TP}{TP+FP}\;,$$

where TP (true positive) and FP (false positive) denote the numbers of correctly and incorrectly predicted connections, respectively. Recall is the ratio of inferred connections among the true connections in $G\left(V,A\right)$:

(2) $$\mathrm{Recall}=\frac{TP}{TP+FN}\;,$$

where FN (false negative) means the number of non-inferred connections in $G\left(V,A\right)$. Structural accuracy is the ratio of correct predictions, as follows:

(3) $$\mathrm{Structural}\;\mathrm{Accuracy}=\frac{TP+TN}{TP+FP+FN+TN}\;,$$

where TN (true negative) is the number of correct negative predictions.

The second performance type is the network dynamics accuracy by comparing E and $E^{'}$. Let $e=e_{1}e_{2}\cdots e_{N}\;(e_{i}\in \{0,1\})$ a steady-state Boolean expression in E and $\alpha_{e}=\left[v\left(1\right),v\left(2\right),\;\ldots ,v\left(p\right)\right]$ the attractor in $E^{'}$ corresponding to e (p is the attractor length). For convenience, we first define the similarity of ith gene between e and$\;\alpha_{e}$ as follows:

$$s_{i}\left(e,\alpha_{e}\right)=\frac{1}{p}\sum_{t=1}^{p}I\left(e_{i}=v_{i}\left(t\right)\right),$$

where $v_{i}\left(t\right)$ means the state value of ith gene in $v\left(t\right)$ and $I\left(\cdot \right)$ is an indicator function that returns 1 if the condition is true, or 0 otherwise. Then the similarity function between e and α denoted by $s(e,\alpha_{e})$ means the average similarity of all genes between e and$\;\alpha_{e}$ as follows:

$$s\left(e,\alpha_{e}\right)=\frac{1}{N}\sum_{i=1}^{N}s_{i}\left(e,\alpha_{e}\right).$$

Then, we evaluate the network dynamics performance of the inferred network using a dynamics accuracy function defined as follows:

(4) $$\mathrm{Dynamics}\;\mathrm{Accuracy}=\frac{1}{\left|E\right|}\sum_{e\in E}s\left(e,\alpha_{e}\right)$$

In other words, the network dynamics accuracy represents the average similarity between the steady-state Boolean expression set (E) and the attractor set $\left(E^{'}\right)$ of the inferred network. We also note that most previous studies have focused on the structural inference performance larger than the dynamics one (Maucher *et al.*, 2011), which is considerably crucial, though, because the network inference ultimately aims to characterize various cellular dynamical behaviors through complex molecular interactions.

In our proposed method, it is necessary to assess how accurately the inferred update function of each target gene fits the steady-state expression dataset (see section ‘A constrained Genetic Algorithm for Boolean network inference’ for details). In this regard, we define the average similarity of ith gene over all experiments as follows:

(5) $$\overset{-}{s}\left(i\right)=\frac{1}{\left|E\right|}\sum_{e\in E}s_{i}\left(e,\alpha_{e}\right)\;.$$

### 2.3 Overall framework of the proposed Boolean network inference algorithm

In this work, we propose a constrained genetic algorithm-based Boolean network inference method called CGA-BNI. We first introduce a procedure to generate path consistency-based constraints from steady-state gene expression data. The set of constraints as well as the steady-state expressions are used as input for our genetic algorithm to infer the Boolean network.

#### 2.3.1 Path consistency-based constraints

A path-consistency constraint can efficiently reduce the cost in searching a feasible network structure. In this study, we devised two measures to establish a path-consistency constraint between a pair of genes using the steady-state expression data. The first measure is the gene–gene coherency which is deduced by comparing the gene expressions between the wild-type and the mutant experiments. Consider a pair of a perturbation experimental Boolean expression vector $e=e_{1}e_{2}\cdots e_{N}\;(e\in {\left\{0,1\right\}}^{N})$ and a corresponding wild-type experimental Boolean expression vector $w=w_{1}w_{2}\cdots w_{N}\;(w\in {\left\{0,1\right\}}^{N})$, and let k (k∈{1, …,N}) the index of the mutated gene in e. For every gene index l≠k, the gene–gene coherency is defined as $\Delta \left(k,l\right)=\left(e_{k}-w_{k}\right)\times \left(e_{l}-w_{l}\right)$. A positive or negative value of $\Delta \left(k,l\right)$ means that two genes have a consistent or inconsistent trend of change in the expression level, which eventually implies the positive or negative path from gene $v_{k}$ to gene $v_{l}$. Next, the second measure is the Pearson correlation coefficient to quantify the strength of a linear association between two genes, which is denoted by r(k,l) as follows:

(6) $$r\left(k,l\right)=\frac{\sum_{e\in E}\left(e_{k}-\overset{-}{e_{k}}\right)\times \left(e_{l}-\overset{-}{e_{l}}\right)}{\sqrt{\sum_{e\in E}{\left(e_{k}-\overset{-}{e_{k}}\right)}^{2}}\times \sqrt{\sum_{e\in E}{\left(e_{l}-\overset{-}{e_{l}}\right)}^{2}}}\;,$$

where ${\overset{-}{e}}_{i}=\frac{1}{\left|E\right|}\sum_{e\in E}e_{i}$ denotes the average Boolean expression value of gene $v_{i}$ in the steady-state Boolean expression dataset E. As the linear association of gene $v_{k}$ and $v_{l}$ gets stronger, the value of r(k,l) is closer to either +1 or -1 depending on the sign of the relationship. As shown in Table 1, we established two criteria of conditions to identify the path consistency-based constraint. The first condition is the positiveness condition based on the signs of $\Delta \left(k,l\right)$ and r(k,l). If both of them are positive (or negative), the network is supposed to contain a positive (resp., negative) path from gene $v_{k}$ to gene $v_{l}$. The second condition determines the directness of the path. If the correlation is relatively strong (|r(k,l)|≥β), a direct path is presumed. On the other hand, if the correlation is relatively weak ($\alpha <\left|r(k,l)\right|<\beta$), an indirect path is presumed. We note that the parameters α and β were heuristically set to 0.1 and 0.5, respectively, in this study. As a result, there can be four types of a regulatory path relation between an ordered pair of genes, if applicable, according to the positiveness and the directedness conditions. The set of all identified regulatory paths are used as the path-consistency constraints which the candidate network should meet during the network inference process.

**Table 1.** Path consistency-based constraints in CGA-BNI

		Positiveness condition	
		$\Delta \left(k,l\right)>0$ and r(k,l)>0	$\Delta \left(k,l\right)<0$ and r(k,l)<0
Directedness Condition	$\left|r(k,l)\right|\geq \beta$	Direct and positive	Direct and negative
	$\alpha <\left|r(k,l)\right|<\beta$	Indirect and positive	Indirect and negative

#### 2.3.2 A constrained genetic algorithm for Boolean network inference

**Fig. 2.**
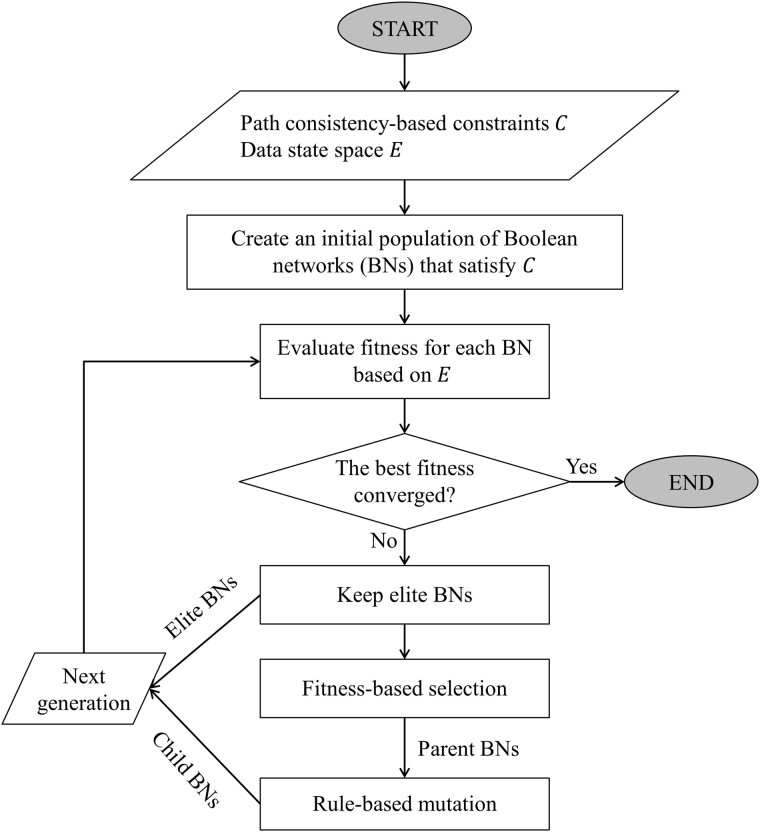
Overall framework of the CGA-BNI algorithm. Our GA takes the path consistency-based constraints C and the observed steady-state data E as inputs and returns a Boolean network such that the topology satisfies C and the induced dynamics is most similar to E. First, an initial population of random feasible Boolean networks (BNs) are generated by using the Barabási-Albert (BA) model (see Supplementary Fig. S1 for the pseudo-code). Some elite BNs are kept in the next population. In addition, the GA selects the parent BNs, produces the new BNs by applying the rule-based mutation operator to the parent BNs, and insert them into the next population. This generation is repeated until a stop condition is met

Our proposed genetic algorithm (GA) takes the set of path-consistency constraints and the Boolean steady-state gene expression as inputs and infers a Boolean network which satisfies the constraints and induces the dynamics as close as to the experimental Boolean gene expression data. The procedure of the proposed method is outlined in Figure 2. Our GA starts with an initial population of 200 random Boolean networks which are generated by using the Barabási-Albert (BA) model (Barabási and Albert, 1999) (see Supplementary Fig. S1 for the pseudo-code) such that the network topology satisfies the path-consistency constraints. Then, the GA creates the next population of networks by conducting the following steps:

- The fitness of each network is scored by the dynamics accuracy (see Equation 4 in Section 2). We note that this task was implemented in parallel by using multi-threaded Java programming where each network is assigned to a Java thread for calculation. This parallel implementation can reduce the computation cost in evaluating a candidate network.
- Some best networks in the current population with higher fitness are chosen as elite, and they are passed to the next population. In this study, we set the number of elite networks to 2.
- A parent network is chosen among the current population. The selection probability of a network is linearly proportional to its fitness.
- A new network is produced by applying the rule-based mutation operator to the parent network.
- The new network replaces the parent network in the population.

The GA stops when the best network in the population is not improved during 200 past generations. We note that our GA did not involve a crossover operator unlike other traditional genetic algorithms. In addition, the path consistency constraints are hard conditions. This is the reason why such mutations that satisfy the path-consistency constraints are allowed in the middle of GA operations. In other words, the constraints must be satisfied by any feasible solution. In the following sub-sections, we introduce the details of our GA including the chromosomes encoding, the selection operation and the mutation operation.

**Chromosomal codification**

In GAs, a solution to a problem is called a chromosome and a population consists of a set of chromosomes. In this study, we represent a chromosome by a set of NCF rules, $\left\{f_{1},f_{2},\ldots ,f_{N}\right\}$ where $f_{i}$ is the Boolean update function for ith gene (see Methods for the definition of NCF). Let $f_{i}$ be a Boolean NCF update function with m regulatory inputs which is represented by the following sequence:

(7) $$f_{i}=\left[\left(v_{i_{1}},I_{1},O_{1}\right),\ldots ,\left(v_{i_{m}},I_{m},O_{m}\right),O_{\mathrm{def}}\right]\;.$$

In addition, we can further divide m tuples in $f_{i}$ into two disjoint subgroups: the fixed-path subgroup (FPS) and the variable-path subgroup (VPS). A tuple is classified into FPS or VPS if it is necessary to meet the path-consistency constraints or not, respectively. For example, a tuple $\left(v_{j},I_{j},O_{j}\right)$ in $f_{i}$ implies the existence of a directed interaction from gene $v_{j}$ to gene $v_{i}$ (Note that the sign of the interaction is determined by the values of $I_{j}$ and $O_{j}$). If $\left(v_{j},I_{j},O_{j}\right)$ is an element of FPS, it is prohibited that the mutation operator in our GA disrupt the path information of the tuple. On the other hand, if it is an element of VPS, there is no restriction about the disruption by the mutation operator.

**Selection**

Our GA selects a parent chromosome among a population to produce a new chromosome for the next population. We adopted the traditional roulette wheel selection scheme where the selection probability of a chromosome x is proportional to the fitness value of x as follows:

(8) $$\mathrm{Pr}\left(x\right)=\frac{\mathrm{fitness}(x)}{\sum_{y\in P}\mathrm{fitness}(y)},$$

where fitness(x) means the dynamics accuracy of x (see Equation 5).

**Mutation**

**Fig. 3.**
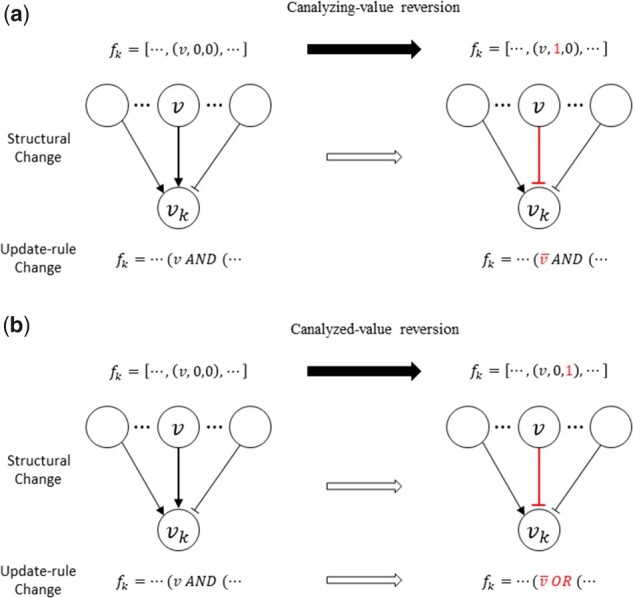
Illustrations of canalyzing/canalyzed-value reversion mutation types in the CGA-BNI algorithm. See Supplementary Figure S2 for other mutations types

In our GA, the mutation operator is used to generate a new chromosome by changing the parent chromosome. Let $\left\{f_{1},f_{2},\ldots ,f_{N}\right\}$ be a parent chromosome selected among the population. Then the mutation operator first randomly chooses one update rule $f_{k}$ such that *i*) the gene-wise dynamics consistency of the gene $v_{k}$ is not perfect (i.e. $\overset{-}{s}\left(k\right)<1$) and *ii*) $\overset{-}{s}\left(k\right)$ is not converged during 100 previous consecutive generations with a 2% tolerance rate. Our GA implemented the following six different types of mutation operators (Fig. 3 and Supplementary Fig. S2 in Supplementary Information), and one of them is randomly applied to the update rule $f_{k}$.

- *Canalyzing-value reversion*: A tuple $\left(v,I,O\right)$ is randomly selected among the VPS of $f_{k}$ and it is replaced by $\left(v,1-I,O\right)$. In other words, this mutation operator flips the corresponding canalyzing value and thus causes to switch the type of molecular interaction between v and $v_{k}$ from a positive interaction to a negative one and vice versa (Fig. 3a).
- *Canalyzed-value reversion*: A tuple $\left(v,I,O\right)$ is randomly selected among the VPS of $f_{k}$ and it is replaced by $\left(v,I,1-O\right)$. In other words, this mutation operator flips the corresponding canalyzed value and thus causes to switch the type of molecular interaction between v and $v_{k}$ from a positive interaction to a negative one and vice versa (Fig. 3b). Although the structural change by this mutation operator is same with that by the canalyzing-value reversion, the difference is that the relationship between v and $v_{k}$ is changed from a conjunction logical function to a disjunction one and vice versa.
- *Canalyzing and canalyzed values reversion*: A tuple $\left(v,I,O\right)$ is randomly selected among the FPS and VPS of $f_{k}$ and it is replaced by $\left(v,1-I,1-O\right)$. This mutation operator flips both the corresponding canalyzing and canalyzed values and thus causes to change only the relationship between v and $v_{k}$ from a conjunction logical function to a disjunction one and vice versa (see Supplementary Fig. S2a in Supplementary Information). We note that this mutation does not affect the type of molecular interaction between v and $v_{k}$.
- *Canalyzing and canalyzed values swapping*: Two FPS tuples or two VPS tuples of $f_{k}$,$\;\left(v,I,O\right)$ and $\left(v^{'},I^{'},O^{'}\right)$, are randomly selected and their orders in the sequence of $f_{k}$ are swapped (see Supplementary Fig. S2b in Supplementary Information). It changes the precedence of two regulatory inputs v and $v^{'}$ in updating the state value of $v_{k}$.
- *Canalyzing and canalyzed values removal*: A tuple $\left(v,I,O\right)$ is randomly selected among the VPS of $f_{k}$ and is removed from $f_{k}$ (see Supplementary Fig. S2c in Supplementary Information). This mutation represents the loss of the molecular interaction between v and $v_{k}$.
- *Canalyzing and canalyzed values insertion*: A new tuple $\left(v,I,O\right)\notin f_{k}$ such that $v\neq v_{k}$ is randomly generated and it is then inserted into the first position in the VPS part of $f_{k}$ (see Supplementary Fig. S2d in Supplementary Information). This mutation represents the gain of a new molecular interaction between v and $v_{k}$. The mutation can be applied only if $\left|r\left(v,v_{k}\right)\right|>\alpha$.

Note that if a chosen mutation operator generates a network which does not meet any path-consistency constraint, it is discarded and another mutation is randomly selected until a feasible network is generated.

## 3 Results

To validate our approach, we tested it with the artificial, the DREAM challenge and a real large-scale gene expression dataset.

### 3.1 Performance on DREAM and artificial datasets

DREAM challenge gives a series of noisy gene expression datasets and gold benchmark networks, which were selected from source networks of real species, *E.coli* and *Yeast*. In this section, we use the steady-state gene expression data from DREAM3 challenge. There are three synthetic datasets: dataset10, dataset50 and dataset100, which contain 10, 50 and 100 genes with variable number of edges, respectively. They express the steady state levels for the wild-type and the null-mutant strains for each gene. The continuous-valued gene expression data was converted to Boolean-valued data by using the *K*-means clustering algorithm (MacQueen, 1967). To show that our method is stable against the Binarization result, we generated 50 different Boolean-valued gene expression datasets by varying the starting centroids in the *K*-means clustering algorithm. Then the Boolean-valued steady-states of all genes were used as an input to execute the CGA-BNI.

In addition, we generated artificial datasets as follows. Ten groups of random Boolean networks with different network sizes ($\left|V\right|=10,\;20,\;\ldots ,\;100$ and $\left|A\right|=2\times \left|V\right|-3$) are created by using the Barabási-Albert (BA) model (Barabási and Albert, 1999) (see Supplementary Fig. S1 in Supplementary Information for the pseudo-code). For each group, 20 networks were generated and thus a total of 200 BA random networks were created. For each network, the update rules of all genes were randomly generated. The number of initial-states was set to 10000, and the corresponding wild-type attractors were computed. In addition, each gene was subject to a knockout perturbation and the corresponding mutated attractor was calculated. In both of DREAM and random BA networks datasets, CGA-BNI outputs an inferred network structure and predicts wild-type and mutated attractors of all genes. Accordingly, we analyzed the performance of CGA-BNI in terms of both structural and dynamics accuracies.

To verify that the inference problem is not trivial, we examined the ratio of ‘0’ value for each gene in one of the binarized steady-state gene expression datasets (see Supplementary Fig. S5 in Supplementary Information). As shown in the figure, most of them are not biased to 0 or 1. This implies that a simple regulatory rule which almost outputs a constant value cannot be a good solution.

**Structural accuracy analysis**

**Fig. 4.**
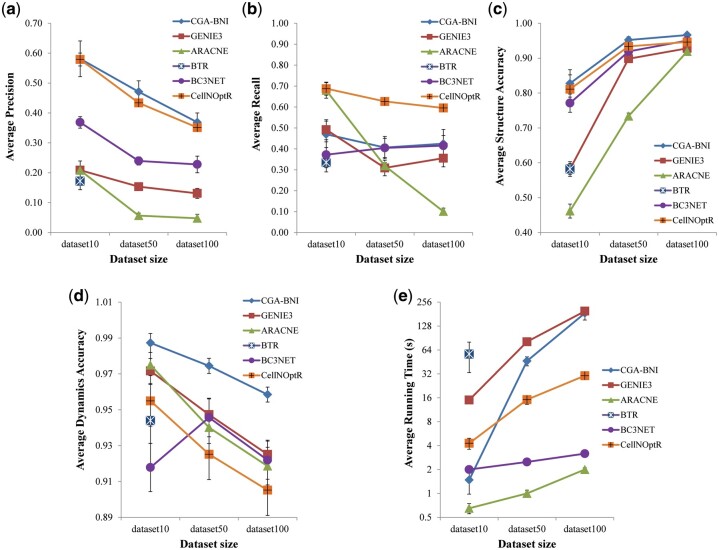
Comparison of precision, recall, structural accuracy, dynamic accuracy and running time between CGA-BNI and other methods in DREAM datasets. (**a**–**e**) Results of precision, recall, structural accuracy, dynamic accuracy and running time, respectively. In all subfigures, the three synthetic datasets (dataset10, dataset50 and dataset100) from DREAM3 challenge with different network sizes $\left|V\right|=10,\;50,\;100$, respectively, were used. Y-axis value and error bar represent the average and 95% confidence interval, respectively

To compare performance, we applied CGA-BNI, CellNOptR, ARACNE, GENIE3, BC3NET and BTR to the DREAM and random BA networks datasets and examined the structural accuracies with respect to the inferred networks (Fig. 4 and Supplementary Fig. S3 in Supplementary Information). CellNOptR is an open-source R package for building predictive logic models of signaling networks by training networks derived from prior knowledge to signaling data (Terfve *et al.*, 2012). ARACNE (Margolin *et al.*, 2006) represents one of the most widely used reverse engineering algorithms, and it uses an information theoretic framework based on the data processing inequality to identify direct regulatory relationships between transcriptional regulator proteins and target genes. GENIE3 (Huynh-Thu *et al.*, 2010) exploits a variable importance score derived from Random Forests to retrieve the regulators of each target gene, and it showed high performance in both the DREAM4 and DREAM5 challenges. BC3NET (de Matos Simoes and Emmert-Streib, 2012) is an ensemble method based on bagging the C3NET algorithm (Altay and Emmert-Streib, 2010) and it is a Bayesian approach with non-informative priors. BTR is an approach based on the swarming hill climbing strategy, in which a population of multiple solutions are heuristically searched in an iterative way until they are converged. We note that both BTR and our method are based on a search strategy in a solution space. However, BTR is more geared toward a local search and it even requires an initial network structure. In fact, BTR was not so scalable that it failed to infer a network in the cases of dataset50 and dataset100 in the DREAM datasets and the cases of the networks with >10 genes in the artificial BA datasets. We used default values for the parameters in all the compared methods.

To achieve a stable performance, we conducted the simulations 50 trials and retrieved the average results of precision, recall and structural accuracy over DREAM (Fig. 4a–c) and BA (Supplementary Fig. S3a–c) in Supplementary Information) datasets. As shown in the Figure 4a and Supplementary Figure S3a, CGA-BNI showed significantly higher precision values than those of the other methods (*P*-values < 0.05), and CellNOptR showed a second-best precision. In terms of recall, CellNOptR was best in both DREAM datasets (Fig. 4b) and BA random datasets (Supplementary Fig. S3b). We note that CellNOptR utilizes priori information about the network structure. All other methods were obviously worse than those three methods in both precision and recall. As a result, CGA-BNI, CellNOptR and BC3NET significantly showed the highest structural accuracy in all datasets (Fig. 4c and Supplementary Fig. S3c). We note that our proposed method showed best performance robustly against the network size. In addition, the binarization parameter has little impact on the performance of our method.

**Dynamics accuracy analysis**

Boolean networks with different structures can produce the same dynamics. In this regard, it is also important to verify the network inference performance in terms of the dynamics accuracy. Therefore, we examined the dynamics accuracy (see Methods section for the definition) of the inferred networks by the CGA-BNI, CellNOptR, ARACNE, GENIE3, BC3NET and BTR methods over the DREAM (Fig. 4d) and random BA networks datasets (Supplementary Fig. S3d in Supplementary Information). We note that the original ARACNE, GENIE3 and BC3NET methods are not available to infer the regulatory functions. In this regard, we added a ‘regulatory-function-search’ routine which tries to find the optimal update function for each gene in the network structure found by those methods (see Supplementary Fig. S4 in Supplementary Information). In fact, it is equivalent to CGA-BNI except that the canalyzing and canalyzed values removal/insertion mutation operations (see Supplementary Fig. S2c and d in Supplementary Information) are excluded from consideration because they will change the network structure. As shown in the Figure 4d and Supplementary Figure S3d, CGA-BNI showed significantly higher dynamics accuracy than all other methods (*P*-values < 0.05). Similar to the structural accuracy, the binarization parameter has little impact on the dynamics accuracy of our method.

**Computation time**

We compared the running time of CGA-BNI with those of the CellNOptR, ARACNE, GENIE3, BC3NET and BTR methods over the DREAM and random BA networks datasets (Fig. 4e and Supplementary Fig. S3e in Supplementary Information). In regard to ARACNE, GENIE3 and BC3NET, we measure the time spent for only finding regulatory interactions (i.e. ‘regulatory-function-search’ execution time was not included). The running time of CGA-BNI was significantly smaller than those of GENIE3 and BTR in both types of datasets. In particular, BTR is another global search algorithm as our method, but it was not applicable for large network of $\left|V\right|>10$. ARACNE and BC3NET achieved the smallest and second-smallest running times in all datasets, but we note that they inferred only the regulatory structure. Despite of the inherently expensive costs, the running time of our method was comparable because the fitness calculation routine in CGA-BNI was implemented in parallel by using the multithreaded Java programming.

### 3.2 Performance on a real gene expression dataset

In order to evaluate the performance of a real expression dataset, we retrieved the largest public *E.coli* microarray dataset (‘*E_coli*_v4_Build_6’) available from the Many Microbe Microarrays database (M3D) (Faith *et al.*, 2008). Among 4297 genes of 446 samples, we extracted 44 knockout/overexpression samples with 32 mutated genes and 11 related wild-type samples (see Supplementary Table S1 in Supplementary Information for details). The real-valued gene expression was converted to the Boolean values using a *K*-means clustering algorithm-based discretization method (MacQueen, 1967). We generated 50 different Boolean-valued gene expression datasets by varying the starting centroids in the *K*-means clustering algorithm. Next, we obtained a list of genes from the RegulonDB database which curates the largest and best-known information on the transcriptional regulation of *E. coli* (Salgado *et al.*, 2013). In this analysis, we focused on the intersection between the M3D and the RegulonDB. Considering a small portion of knockout/overexpression samples, we reduced the network by retaining only out-going interactions of the 32 mutated genes. Consequently, we constructed a *E. coli* network including 925 genes and 1346 transcriptional interactions as a gold standard (see Supplementary Table S2 in Supplementary Information for details). We note that BTR was not applicable for this analysis due to the unfeasible computational time.

As shown in Table 2, CGA-BNI achieved the highest precision and recall values among all methods. With respect to the structural accuracy metric, the number of actual and predicted interactions were less than 2000 among 925 × 924 × 2 possible interactions in the *E. coli* network with 925 genes, and thus the comparison was not effective because of a huge number of true negative cases in inference of a large-scale network. Moreover, CGA-BNI showed significantly higher dynamics accuracy than all the other methods. It seems that our GA search might easily find regulatory rules fitting the expression data. In addition, the small standard deviation means that the performance of our method was stable against the binarization parameter. In regard to the running time, we compared the time to infer both structure and regulatory function. In other words, the execution time of ‘regulatory-function-search’ routine for the cases of GENIE3, ARACNE and BC3NET are also included in the total running time. As shown in the table, CGA-BNI was second slowest among them. ARACNE and BC3NET were fastest but their accuracy performances were not acceptable. Taken together, these results indicate that CGA-BNI method is suitable to infer both regulatory interactions and functions of large-scale GRNs when high accuracy is desired at the cost of sacrificing the running time.

**Table 2.** Comparison of precision, recall, structural accuracy, dynamic accuracy and running time between CGA-BNI and other methods in the large-scale *E.coli* expression dataset. Bold face means the best result in the corresponding metric.

Method	Precision	Recall	Structure accuracy	Dynamic accuracy	Running time(s)
CGA-BNI	**0.254 ± 0.001**	**0.333 ± 0.000**	**0.997 ± 0.000**	**0.806 ± 0.001**	2614 ± 145
CellNOptR	0.230 ± 0.001	0.293 ± 0.001	0.995 ± 0.000	0.685 ± 0.000	2439 ± 164
GENIE3	0.006 ± 0.000	0.018 ± 0.001	0.994 ± 0.000	0.614 ± 0.001	7454 ± 1003
ARACNE	0.005 ± 0.000	0.015 ± 0.000	0.994 ± 0.000	0.602 ± 0.000	232±13
BC3NET	0.006 ± 0.001	0.015 ± 0.001	0.995 ± 0.000	0.604 ± 0.001	**131±6**

## 4 Conclusions

In this study, we proposed a novel constrained genetic algorithm-based Boolean network inference method, CGA-BNI. Through extensive simulations on the benchmark datasets from DREAM challenge, artificial datasets and a large-scale gene expression dataset in *E. coli*, CGA-BNI showed consistently better performance than four well-known existing methods, ARACNE, GENIE3, BC3NET and BTR in terms of both structural and dynamics prediction accuracy. It turns out that the GA search combining with path consistency-based constraints and network dynamics is efficient to infer a network from gene expression data. CGA-BNI also showed acceptable running time for the large-scale dataset. Taken together, CGA-BNI is a promising tool for predicting both the structure and the dynamics of a gene regulatory network.

There are some notable issues to be discussed. First, we used the synchronous update scheme for the Boolean network model. In fact, it is more likely that the genes are asynchronously updated in the real signaling networks. However, the asynchronous update scheme requires some additional parameters such as the update order of genes, which are generally unknown. Considering that they affect the network dynamics greatly, it is not guaranteed that the asynchronous update scheme can describe the dynamics of a real signaling network more accurately than the synchronous scheme. In addition, we employed NCFs to randomly specify the update rule. It is known that NCFs can represent various types of regulatory interactions (Samal and Jain, 2008; Trinh and Kwon, 2016). Despite of the effectiveness of NCFs, a more realistic representation model of the regulatory interaction can improve the usefulness of our tool. Another issue is that CGA-BNI can include a way to help the search to avoid local optima. In fact, it already has some characteristics to keep the diversity of a population such as a relatively large population size, no crossover operation and the replacement of a parent solution with a worse new solution. Finally, it is necessary to validate our approach through other evaluation framework using a single-cell transcriptomic data (Pratapa *et al.*, 2020).

## Funding

This work was supported by the 2021 Research Fund of University of Ulsan.

*Conflict of Interest*: none declared.

## Supplementary Material

btab295_Supplementary_Data
