# Supplementary material for: A novel constrained genetic algorithm-based Boolean network inference method from steady-state gene expression data
Source: Bioinformatics. 2021 Jul 12;37(Suppl 1):i383–91. doi: 10.1093/bioinformatics/btab295 (PMC8275338; doi:10.1093/bioinformatics/btab295)
Supplement: btab295_Supplementary_Data [file btab295_supplementary_data.pdf]

A novel constrained genetic algorithm-based Boolean network inference method from steady-state gene expression data

Hung-Cuong Trinh [1] and Yung-Keun Kwon [2,*]

[1] Faculty of Information Technology, Ton Duc Thang University, Ho Chi Minh City, Vietnam

[2] Department of Electrical/Electronic and Computer Engineering, University of Ulsan, 93 Daehak-ro, Nam-gu, Ulsan 44610, Korea

[*]Corresponding author

E-mail: kwonyk@ulsan.ac.kr

Hung-Cuong Trinh

E-mail: trinhhungcuong@tdtu.edu.vn

Supporting Information

Supplementary Figures and Tables

```
function [V, A] = createRBN_BarabasiAlbert(N, e, d)
    //N:          The number of desirable nodes
    //e:          The number of initial nodes
    //d:          The number of interactions added at each step
    //V, A:       A set of nodes V and a set of links A of the resulting network generated by the Barabási-Albert model

    V←{0, 1, …, e-1};
    A←∅;

    for i:=0 to e-2
        for j:=i+1 to e-1
          if (randNumber(0,1) < 0.5)  // randNumber(0,1) returns a real number chosen from 0 to 1 uniformly at random
              A←A∪{(i, j)};
          else
              A←A∪{(j, i)};
          endif
        endfor
    endfor

    for i:= e to N-1
        for j:=0 to d-1
          do
              v = selection(V); //v is chosen with a probability proportional to its degree.
              if (randNumber(0,1) < 0.5)
                  vSrc← i, vDst← v;
              else
                  vSrc← v, vDst← i;
              endif
          until ((vSrc, vDst)∉ A);

          A←A∪{(vSrc, vDst)};
        endfor

        V←V∪{i};
    endfor

    return [V, A];
end
```

Figure S1. Pseudo-code for the Barabási-Albert model.

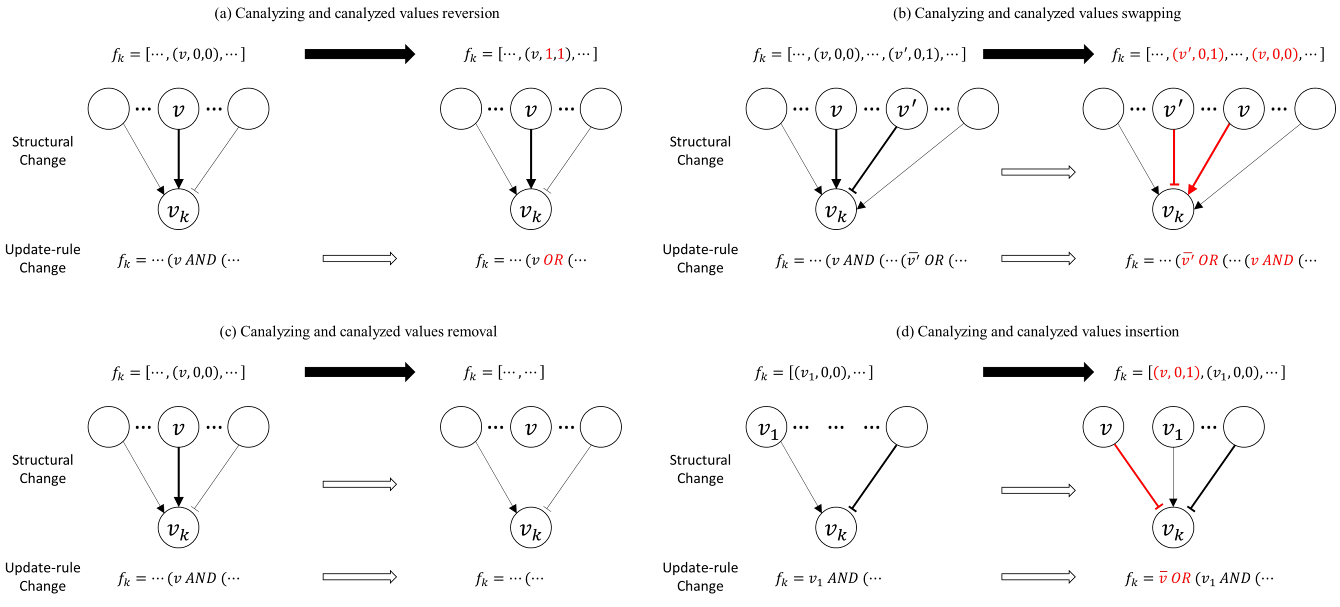

Figure S2. Illustrations of four other mutation types in the CGA-BNI algorithm.

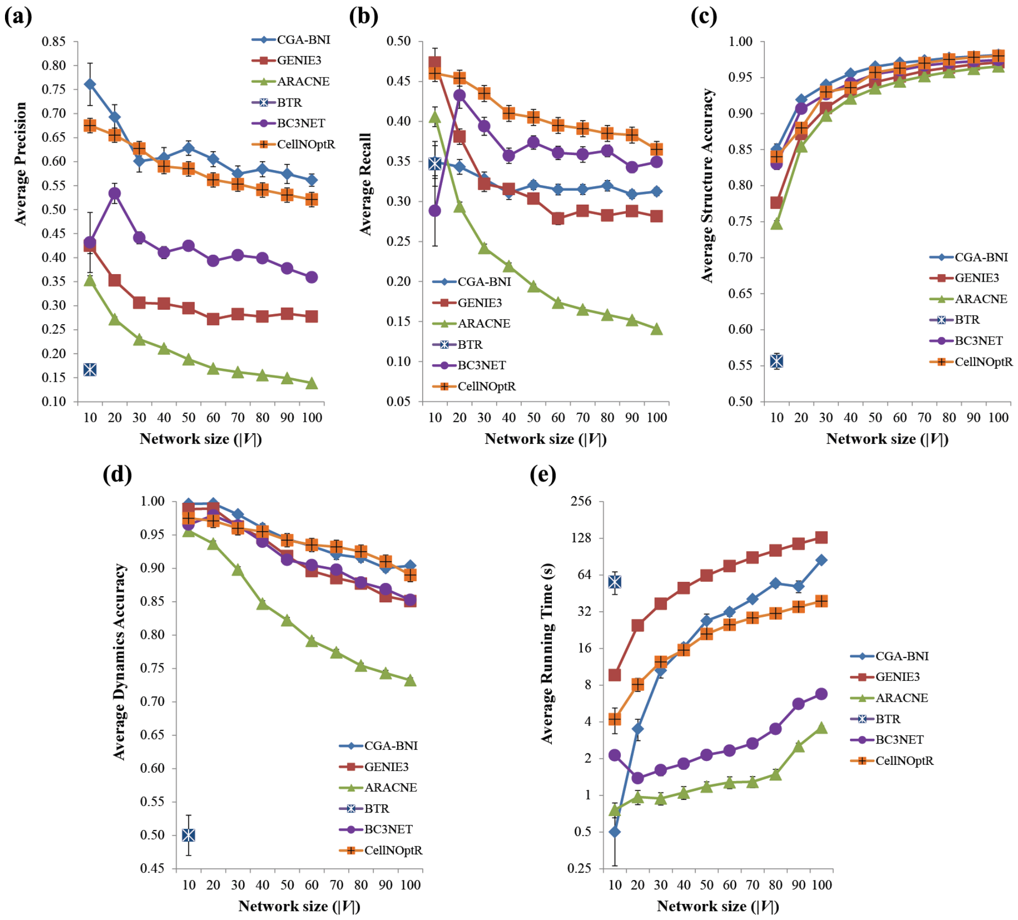

Figure S3. Comparison of precision, recall, structural accuracy, dynamic accuracy, and running time between CGA-BNI and other methods in random BA networks datasets. (a)-(e) Results of precision, recall, structural accuracy, dynamic accuracy, and running time, respectively. In all subfigures, ten groups of random Boolean networks with different network sizes ($|V| = 10, 20, \ldots, 100$ and $|A| = 2 \times |V| - 3$) are created by using the BA model. Y-axis value and error bar represent the average and 95% confidence interval, respectively.

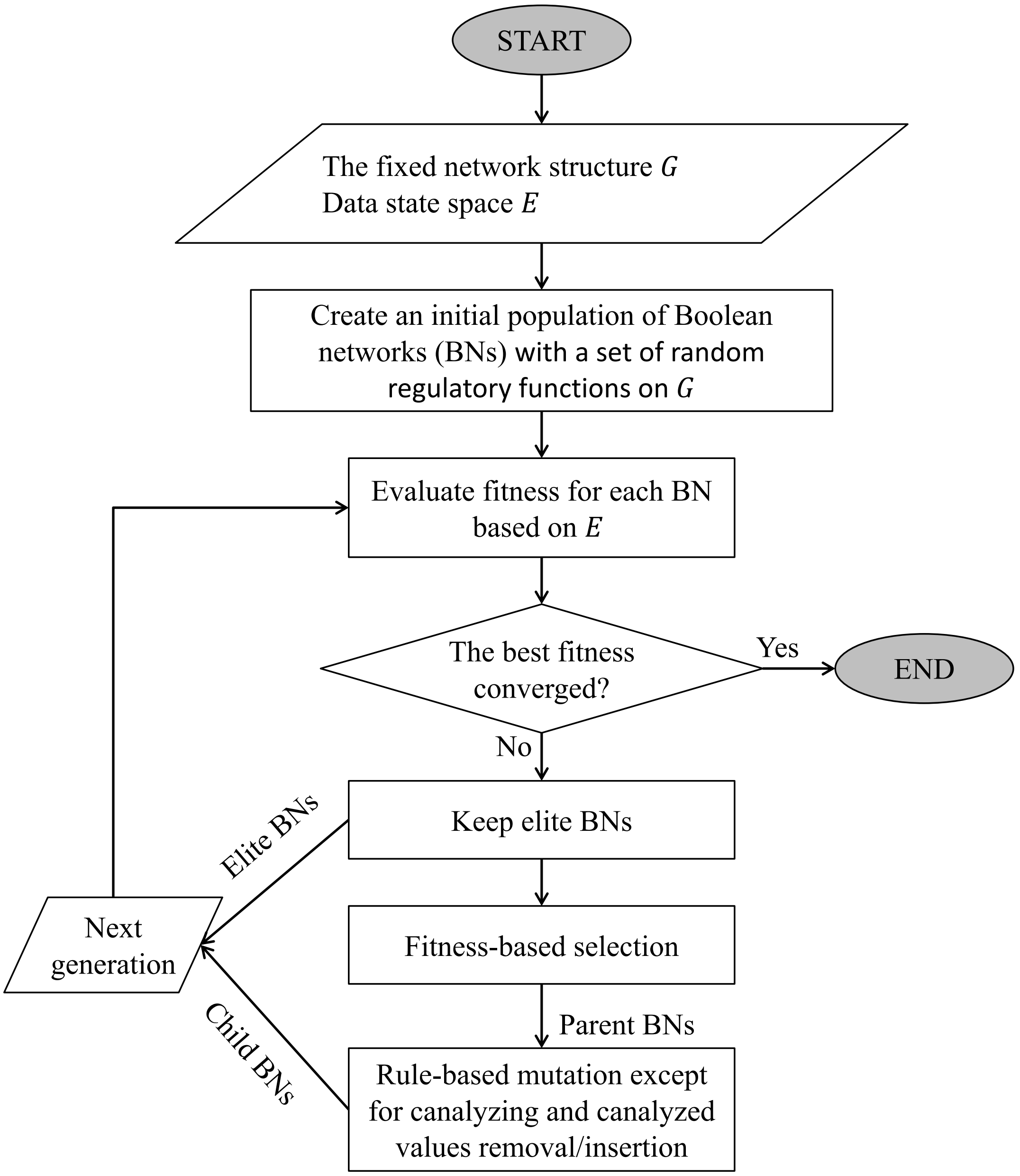

Figure S4. Overall framework of the regulatory-function-search routine. The routine is used to search a set of the regulatory functions which can best fit the expression dataset on a network with a fixed network structure. In this work, the network structure is determined by the inference method such as ARACNE, GENIE3, BC3NET, or BTR, which can infer only the network structure. Similar to CGA-BNI, it uses the genetic algorithm framework. The initial population of Boolean networks are created by randomly generating a set of regulatory functions for the given network structure. We note that the canalyzing and canalyzed values removal/insertion mutation operations are not taken into account unlike CGA-BANI because they may change the network structure.

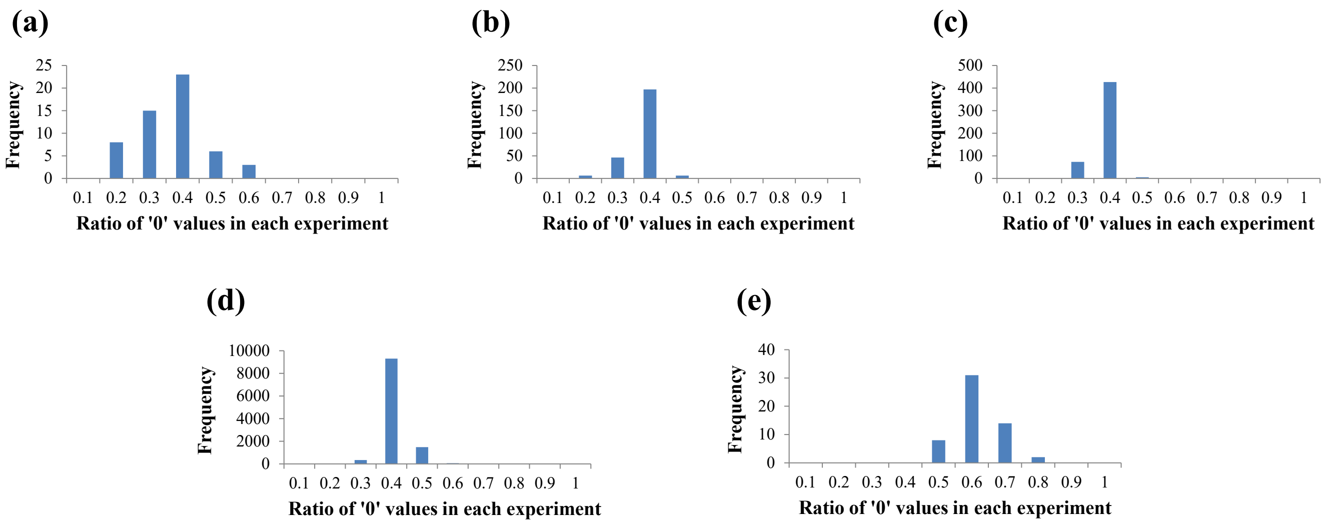

Figure S5. Ratio of '0' values in each experiment from DREAM datasets, random BA networks, and the large-scale E.coli expression datasets. (a)-(c) Results of three synthetic datasets, dataset10, dataset50 and dataset100 from DREAM3 challenge with different network sizes |V|=10,50,100, respectively. (d) Results of random BA networks datasets. Ten groups of random Boolean networks with different network sizes (|V|=10,20,…,100 and |A|=2×|V|-3) are created by using the BA model. (e) Results of the large-scale E.coli expression dataset (including 925 genes and 1346 transcriptional interactions).

Table S1. List of the knockout/overexpression samples and related wild-type samples.

experiment_name	perturbation	perturbation_gene	Wild-type
dinI_U_N0025	over_expression	dinI	WT_N0025
lexA_U_N0025	over_expression	lexA	WT_N0025
lon_U_N0025	over_expression	lon	WT_N0025
recA_U_N0025	over_expression	recA	WT_N0025
relA_U_N0025	over_expression	relA	WT_N0025
ruvA_U_N0025	over_expression	ruvA	WT_N0025
sulA_U_N0025	over_expression	sulA	WT_N0025
umuD_U_N0025	over_expression	umuD	WT_N0025
uvrA_U_N0025	over_expression	uvrA	WT_N0025
cpxR_U_N0075	over_expression	cpxR	WT_N0075
crp_U_N0075	over_expression	crp	WT_N0075
dnaA_U_N0075	over_expression	dnaA	WT_N0075
dnaN_U_N0075	over_expression	dnaN	WT_N0075
fis_U_N0075	over_expression	fis	WT_N0075
folA_U_N0075	over_expression	folA	WT_N0075
gyrA_U_N0075	over_expression	gyrA	WT_N0075
murI_U_N0075	over_expression	murI	WT_N0075
nrdA_U_N0075	over_expression	nrdA	WT_N0075
nrdB_U_N0075	over_expression	nrdB	WT_N0075
nupC_U_N0075	over_expression	nupC	WT_N0075
pyrC_U_N0075	over_expression	pyrC	WT_N0075
rstB_U_N0075	over_expression	rstB	WT_N0075
uspA_U_N0075	over_expression	uspA	WT_N0075
recA_D_N0000	knockout	recA	WT_N0000
recA_D_N0050	knockout	recA	WT_N0050
recA_D_N0100	knockout	recA	WT_D_N0100
M9_K_appY	knockout	appY	M9_WT
M9_K_arcA	knockout	arcA	M9_WT
M9_K_fnr	knockout	fnr	M9_WT
M9_K_oxyR	knockout	oxyR	M9_WT
M9_K_soxS	knockout	soxS	M9_WT
M9_K_appY_anaerobic	knockout	appY	M9_WT_anaerobic
M9_K_arcA_anaerobic	knockout	arcA	M9_WT_anaerobic
M9_K_fnr_anaerobic	knockout	fnr	M9_WT_anaerobic
M9_K_oxyR_anaerobic	knockout	oxyR	M9_WT_anaerobic
M9_K_soxS_anaerobic	knockout	soxS	M9_WT_anaerobic
MOPS_K_crp	knockout	crp	WT_MOPS_glucose
MOPS_K_cspA	knockout	cspA	WT_MOPS_glucose
MOPS_K_dps	knockout	dps	WT_MOPS_glucose
MOPS_K_dps_stationary	knockout	dps	WT_MOPS_stationary
MOPS_K_dps_stationary2	knockout	dps	WT_MOPS_stationary2
MOPS_K_hns	knockout	hns	WT_MOPS_glucose
MOPS_K_hupB	knockout	hupB	WT_MOPS_glucose
fnr_K_fnrAnaerobic	knockout	fnr	fnr_wtAnaerobic

Table S2. Gold standard *E. coli* network with 925 genes and 1346 transcriptional interactions.

Transcription factor	Regulated gene	Interaction Type
oxyr	fuci	+
oxyr	gabd	+
oxyr	dps	+
oxyr	dsbg	+
oxyr	fhuf	-
oxyr	flu	-
oxyr	fur	+
oxyr	gntp	-
oxyr	gor	+
oxyr	grxa	+
oxyr	hcp	+
oxyr	hcr	+
oxyr	hemh	+
oxyr	isrc	-
oxyr	katg	+
oxyr	mnth	+
oxyr	nfsa	-
oxyr	oxys	+
oxyr	rimk	-
oxyr	sufa	+
oxyr	sufb	+
oxyr	sufc	+
oxyr	sufd	+
oxyr	sufe	+
oxyr	sufs	+
oxyr	trxc	+
oxyr	uof	+
oxyr	uxua	-
oxyr	uxub	-
oxyr	ybjc	-
oxyr	ybjn	-
oxyr	ychf	-
oxyr	yhja	+
soxs	#N/A	+
soxs	cspd	+
soxs	cyoa	+
soxs	flda	+
soxs	fldb	+
soxs	fpr	+
soxs	fumc	+
soxs	fur	+
soxs	inaa	+
soxs	mara	+
soxs	marb	+
soxs	marr	+
soxs	micf	+
soxs	nfo	+

soxs	nfsa	+
soxs	nfsb	+
soxs	ompn	+
soxs	pgi	+
soxs	poxb	+
soxs	pqia	+
soxs	pqib	+
soxs	ptsg	+
soxs	riba	+
soxs	rimk	+
soxs	rob	-
soxs	soda	+
soxs	tolc	+
soxs	uof	+
soxs	ybjc	+
soxs	ybjn	+
soxs	ydbk	+
soxs	ygib	+
soxs	ygic	+
soxs	zwf	+
cspa	gyra	+
cspa	hns	+
dnaa	galk	-
dnaa	dnan	-
dnaa	guaa	-
dnaa	guab	-
dnaa	nrda	+
dnaa	nrda	-
dnaa	nrdb	+
dnaa	nrdb	-
dnaa	pola	+
dnaa	recf	-
dnaa	rpoh	-
dnaa	yfae	+
dnaa	yfae	-
fnr	rpmc	-
fnr	tolc	-
fnr	#N/A	+
fnr	#N/A	-
fnr	dinb	+
fnr	dmsc	+
fnr	epd	+
fnr	fadl	+
fnr	galk	-
fnr	glpf	+
fnr	mhpe	+
fnr	mhpe	-
fnr	nrff	+
fnr	paaf	+

fnr	pflb	+
fnr	rrsh	+
fnr	rutg	+
fnr	sera	+
fnr	sohb	+
fnr	srle	+
fnr	sucb	+
fnr	sxy	+
fnr	tdcf	+
fnr	uvrb	+
fnr	xdha	+
fnr	yaep	+
fnr	ybfn	+
fnr	ydea	+
fnr	yebe	+
fnr	ygic	+
fnr	yidq	+
fnr	#N/A	+
fnr	#N/A	+
fnr	#N/A	+
fnr	#N/A	+
fnr	#N/A	-
fnr	#N/A	-
fnr	#N/A	-
fnr	#N/A	-
fnr	#N/A	-
fnr	#N/A	-
fnr	cyda	+
fnr	cyda	-
fnr	cydb	+
fnr	cydb	-
fnr	cydc	+
fnr	cydd	+
fnr	cyoa	-
fnr	cyob	-
fnr	cyoc	-
fnr	cyod	-
fnr	cyoe	-
fnr	cysg	+
fnr	dcua	+
fnr	dcub	+
fnr	dcuc	+
fnr	dcur	+
fnr	dcus	+
fnr	dmsa	+
fnr	dmsb	+
fnr	dmsc	+
fnr	dmsd	+
fnr	dpia	-

fnr	dpib	-
fnr	dppa	-
fnr	dppb	-
fnr	dppc	-
fnr	dppd	-
fnr	dppf	-
fnr	emrk	+
fnr	emry	+
fnr	entf	+
fnr	fdhf	+
fnr	fdng	+
fnr	fdnh	+
fnr	fdni	+
fnr	feoa	+
fnr	feob	+
fnr	feoc	+
fnr	fepe	+
fnr	fes	+
fnr	fhla	+
fnr	fixa	+
fnr	fixb	+
fnr	fixc	+
fnr	fixx	+
fnr	foca	+
fnr	focb	-
fnr	frda	+
fnr	frdb	+
fnr	frdc	+
fnr	frdd	+
fnr	fuma	-
fnr	fumb	+
fnr	fumc	-
fnr	gada	-
fnr	gadw	-
fnr	gadx	-
fnr	gark	+
fnr	garl	+
fnr	garp	+
fnr	garr	+
fnr	gcvh	+
fnr	gcvp	+
fnr	gcvt	+
fnr	gdha	+
fnr	glpa	+
fnr	glpb	+
fnr	glpc	+
fnr	glpq	+
fnr	glpt	+
fnr	gltb	-

fnr	gltd	-
fnr	gltf	-
fnr	gnd	-
fnr	hcp	+
fnr	hcr	+
fnr	hema	-
fnr	hlye	+
fnr	hmp	-
fnr	hyfa	-
fnr	hyfb	-
fnr	hyfc	-
fnr	hyfd	-
fnr	hyfe	-
fnr	hyff	-
fnr	hyfg	-
fnr	hyfh	-
fnr	hyfi	-
fnr	hyfj	-
fnr	hyfr	-
fnr	hypb	+
fnr	hypc	+
fnr	hypd	+
fnr	hype	+
fnr	katg	+
fnr	malp	+
fnr	malq	+
fnr	moaa	+
fnr	moab	+
fnr	moac	+
fnr	moad	+
fnr	moae	+
fnr	moea	-
fnr	moeb	-
fnr	nac	-
fnr	napa	+
fnr	napb	+
fnr	napc	+
fnr	napd	+
fnr	napf	+
fnr	napg	+
fnr	naph	+
fnr	narg	+
fnr	narh	+
fnr	nari	+
fnr	narj	+
fnr	nark	+
fnr	narl	-
fnr	narx	-
fnr	ndh	-

fnr	nika	+
fnr	nikb	+
fnr	nikc	+
fnr	nikd	+
fnr	nike	+
fnr	nikr	+
fnr	nirb	+
fnr	nirc	+
fnr	nird	+
fnr	norv	-
fnr	norw	-
fnr	nrdd	+
fnr	nrdg	+
fnr	nrfa	+
fnr	nrfb	+
fnr	nrfc	+
fnr	nrfd	+
fnr	nrfe	+
fnr	nrff	+
fnr	nrfg	+
fnr	nuoa	-
fnr	nuob	-
fnr	nuoc	-
fnr	nuoe	-
fnr	nuof	-
fnr	nuog	-
fnr	nuoh	-
fnr	nuoi	-
fnr	nuoj	-
fnr	nuok	-
fnr	nuol	-
fnr	nuom	-
fnr	nuon	-
fnr	ompw	+
fnr	ompw	-
fnr	ompx	-
fnr	pept	+
fnr	pflb	+
fnr	phem	-
fnr	phou	+
fnr	pita	+
fnr	prfa	-
fnr	prmc	-
fnr	psta	+
fnr	pstb	+
fnr	pstc	+
fnr	psts	+
fnr	purm	-
fnr	purn	-

fnr	puua	-
fnr	puup	-
fnr	rimm	-
fnr	rnpb	+
fnr	rplb	+
fnr	rplc	+
fnr	rpld	+
fnr	rplm	-
fnr	rplp	+
fnr	rpls	-
fnr	rplt	-
fnr	rplv	+
fnr	rplw	+
fnr	rpmc	+
fnr	rpsc	+
fnr	rpsi	-
fnr	rpsj	+
fnr	rpsp	-
fnr	rpsq	+
fnr	rpss	+
fnr	sdha	-
fnr	sdhb	-
fnr	sdhc	-
fnr	sdhd	-
fnr	soda	-
fnr	soxr	-
fnr	soxs	-
fnr	ssua	+
fnr	ssub	+
fnr	ssuc	+
fnr	ssud	+
fnr	ssue	+
fnr	suca	-
fnr	sucb	-
fnr	succ	-
fnr	sucd	-
fnr	tap	+
fnr	tar	+
fnr	tdca	+
fnr	tdcb	+
fnr	tdcc	+
fnr	tdcd	+
fnr	tdce	+
fnr	tdcf	+
fnr	tdcg	+
fnr	tpx	-
fnr	trmd	-
fnr	ubia	-
fnr	ubic	-

fnr	upp	+
fnr	uraa	+
fnr	uxaa	+
fnr	uxac	+
fnr	xdha	+
fnr	xdhb	+
fnr	xdhc	+
fnr	ybdn	+
fnr	ybdz	+
fnr	ycho	-
fnr	ydej	+
fnr	ydht	+
fnr	ydhu	+
fnr	ydhv	+
fnr	ydhw	+
fnr	ydhx	+
fnr	ydhy	+
fnr	yecr	+
fnr	yeil	-
fnr	ygba	-
fnr	yhja	+
fnr	ynfe	+
fnr	ynff	+
fnr	ynfg	+
fnr	ynfh	+
fnr	yqji	+
fnr	ysga	-
fnr	ytfe	-
fnr	zwf	-
fis	csga	-
fis	dppd	-
fis	ebgc	-
fis	epd	+
fis	gata	-
fis	glpf	-
fis	gltf	+
fis	gor	+
fis	mtla	+
fis	nac	+
fis	quea	-
fis	reca	-
fis	rhar	-
fis	tolc	+
fis	trxa	+
fis	ugpc	-
fis	ulad	-
fis	crp	-
fis	cspa	+
fis	cspi	-

fis	cyoa	+
fis	cyob	+
fis	cyoc	+
fis	cyod	+
fis	cyoe	+
fis	cysg	-
fis	deoa	+
fis	deob	+
fis	deoc	+
fis	deod	+
fis	dmsa	-
fis	dmsb	-
fis	dmsc	-
fis	dps	-
fis	dusb	-
fis	fada	+
fis	fadb	+
fis	flxa	+
fis	fumb	-
fis	gada	-
fis	gadb	-
fis	gadc	-
fis	gadx	-
fis	glcc	-
fis	glna	+
fis	glng	+
fis	glnl	+
fis	glnq	-
fis	glpa	+
fis	glpb	+
fis	glpc	+
fis	glpq	+
fis	glpt	+
fis	gltx	-
fis	glyt	+
fis	glyu	+
fis	guaa	-
fis	guab	-
fis	gyra	-
fis	gyrb	-
fis	hisr	+
fis	hlye	-
fis	hns	+
fis	hupa	+
fis	hupb	-
fis	hyaa	-
fis	hyab	-
fis	hyac	-
fis	hyad	-

fis	hyae	-
fis	hyaf	-
fis	infb	+
fis	kate	-
fis	leup	+
fis	leux	+
fis	lpd	+
fis	male	+
fis	malf	+
fis	malg	+
fis	mara	+
fis	marb	+
fis	marr	+
fis	mazg	+
fis	mety	+
fis	mgla	-
fis	mglc	-
fis	msra	-
fis	mtla	-
fis	mtld	-
fis	mtlr	-
fis	nana	-
fis	nane	-
fis	nank	-
fis	nant	-
fis	narg	-
fis	narh	-
fis	nari	-
fis	narj	-
fis	nark	-
fis	ndh	+
fis	ndh	-
fis	nirb	-
fis	nirc	-
fis	nird	-
fis	nrda	+
fis	nrdb	+
fis	nrfa	-
fis	nrfb	-
fis	nrfc	-
fis	nrfd	-
fis	nrfe	-
fis	nrff	-
fis	nrfg	-
fis	nuoa	+
fis	nuob	+
fis	nuoc	+
fis	nuoe	+
fis	nuof	+

fis	nuog	+
fis	nuoh	+
fis	nuoi	+
fis	nuoj	+
fis	nuok	+
fis	nuol	+
fis	nuom	+
fis	nuon	+
fis	nusa	+
fis	ogt	-
fis	osme	-
fis	osmy	-
fis	pdxa	+
fis	pflb	-
fis	pnp	+
fis	prok	+
fis	prol	+
fis	prom	+
fis	prop	+
fis	ptsg	-
fis	pyrd	-
fis	quea	+
fis	rbfa	+
fis	rnpb	+
fis	rnpb	-
fis	rplm	-
fis	rpsi	-
fis	rpso	+
fis	rrfa	+
fis	rrla	+
fis	rrsc	+
fis	rrsg	+
fis	rrsh	+
fis	sra	+
fis	ssrs	+
fis	ssrs	-
fis	thrt	+
fis	thru	+
fis	thrv	+
fis	thrw	+
fis	topa	+
fis	topa	-
fis	trg	+
fis	trma	+
fis	trub	+
fis	tyru	+
fis	xylf	-
fis	xylg	-
fis	xylh	-

fis	xylr	-
fis	year	-
fis	yfae	+
fis	yhch	-
fis	yjch	-
fis	yoag	-
cpxr	dcub	+
cpxr	narl	+
cpxr	nuoj	+
cpxr	ompc	+
cpxr	oppf	+
cpxr	zwf	-
cpxr	#N/A	-
cpxr	cpxa	+
cpxr	cpxp	+
cpxr	csga	-
cpxr	csgb	-
cpxr	csgc	-
cpxr	csgd	-
cpxr	csge	-
cpxr	csgf	-
cpxr	csgg	-
cpxr	cyar	-
cpxr	degp	+
cpxr	dsba	+
cpxr	dsbc	+
cpxr	fabz	+
cpxr	ftnb	+
cpxr	hha	+
cpxr	lpxa	+
cpxr	lpxd	+
cpxr	mara	+
cpxr	marb	+
cpxr	marr	+
cpxr	mdta	+
cpxr	mdtb	+
cpxr	mdtc	+
cpxr	mdtd	+
cpxr	mota	-
cpxr	motb	-
cpxr	ompc	+
cpxr	ompf	-
cpxr	ppia	+
cpxr	ppid	+
cpxr	psd	-
cpxr	rdoa	+
cpxr	rpoe	-
cpxr	rpoh	+
cpxr	rpra	+

cpxr	rsea	-
cpxr	rseb	-
cpxr	rsec	-
cpxr	sbma	+
cpxr	skp	+
cpxr	slt	+
cpxr	spy	+
cpxr	tomb	+
cpxr	tsr	-
cpxr	ung	-
cpxr	yaiw	+
cpxr	ycca	+
cpxr	yebe	+
cpxr	yidq	+
cpxr	yqae	+
cpxr	yqja	+
hybb	hcp	+
hybb	hlye	+
hybb	hpt	+
hybb	hyaa	+
hybb	hyab	+
hybb	hyac	+
hybb	hyad	+
hybb	hyae	+
hybb	hyaf	+
lexa	#N/A	-
lexa	ddlb	-
lexa	dinb	-
lexa	dind	-
lexa	dinf	-
lexa	ding	-
lexa	dini	-
lexa	dinj	-
lexa	dinq	-
lexa	dnag	-
lexa	ftsa	-
lexa	ftsi	-
lexa	ftsk	-
lexa	ftsl	-
lexa	ftsq	-
lexa	ftsw	-
lexa	ftsz	-
lexa	hoke	-
lexa	insk	-
lexa	lpxc	-
lexa	mray	-
lexa	murc	-
lexa	murd	-
lexa	mure	-

lexa	murf	-
lexa	murg	-
lexa	phr	-
lexa	polb	-
lexa	ptra	-
lexa	reca	-
lexa	recb	-
lexa	recd	-
lexa	recn	-
lexa	recx	-
lexa	rpod	-
lexa	rpsu	-
lexa	ruva	-
lexa	ruvb	-
lexa	sbmc	-
lexa	ssb	-
lexa	sula	-
lexa	syme	-
lexa	tisb	-
lexa	umuc	-
lexa	umud	-
lexa	uvra	-
lexa	uvrb	-
lexa	uvrc	-
lexa	uvrd	-
lexa	uvry	-
lexa	yafn	-
lexa	yafo	-
lexa	yafp	-
lexa	yafq	-
lexa	ybfe	-
lexa	ydjm	-
lexa	yebg	-
crp	#N/A	+
crp	epd	+
crp	gdha	-
crp	hypd	+
crp	mtla	-
crp	paaf	-
crp	rpmc	+
crp	tolc	+
crp	ygic	-
crp	#N/A	+
crp	csga	+
crp	dppd	+
crp	ebgc	+
crp	fadl	+
crp	fecc	+
crp	fhuf	+

crp	flhd	+
crp	frdd	+
crp	galk	+
crp	gata	+
crp	gdha	-
crp	glgs	-
crp	glpf	+
crp	hyfd	+
crp	hypd	+
crp	inaa	+
crp	lacy	+
crp	lpxa	+
crp	lsrr	+
crp	malt	+
crp	mazg	+
crp	melr	+
crp	melr	-
crp	moda	+
crp	napc	-
crp	nikc	+
crp	norw	+
crp	nrff	+
crp	quea	+
crp	reca	+
crp	rhar	+
crp	rplb	-
crp	rutg	+
crp	sera	+
crp	sohb	+
crp	srle	+
crp	sucb	+
crp	sxy	+
crp	tdcf	+
crp	yqja	+
crp	yqja	-
crp	zwf	+
crp	zwf	+
crp	zwf	+
crp	zwf	+
crp	zwf	+
crp	zwf	+
crp	#N/A	+
crp	#N/A	-
crp	#N/A	-
crp	#N/A	-
crp	#N/A	-
crp	#N/A	-
crp	#N/A	-
crp	#N/A	+

crp	crr	+
crp	crr	-
crp	csgd	+
crp	csge	+
crp	csgf	+
crp	csgg	+
crp	csid	+
crp	csie	+
crp	cspd	+
crp	cspe	+
crp	csta	+
crp	cyaa	-
crp	cyar	+
crp	cyoa	+
crp	cyob	+
crp	cyoc	+
crp	cyod	+
crp	cyoe	+
crp	cysg	-
crp	dada	+
crp	dadx	+
crp	dcta	+
crp	dcua	+
crp	dcub	+
crp	dcur	+
crp	deoa	+
crp	deob	+
crp	deoc	+
crp	deod	+
crp	dksa	+
crp	dpia	-
crp	dpib	-
crp	dsda	+
crp	dsdx	+
crp	dusb	+
crp	ebga	+
crp	ebgc	+
crp	enta	+
crp	entb	+
crp	entc	+
crp	entd	+
crp	ente	+
crp	envz	+
crp	envz	-
crp	epd	+
crp	exut	+
crp	fadd	+
crp	fadh	+
crp	fadl	+

crp	fbaa	+
crp	feab	+
crp	fear	+
crp	feca	+
crp	fecb	+
crp	fecc	+
crp	fecd	+
crp	fece	+
crp	fepa	+
crp	fis	+
crp	fiu	+
crp	fixa	+
crp	fixb	+
crp	fixc	+
crp	fixx	+
crp	flhc	+
crp	flhd	+
crp	foca	+
crp	focb	+
crp	fuca	+
crp	fuci	+
crp	fuck	+
crp	fuco	+
crp	fucp	+
crp	fucr	+
crp	fucu	+
crp	fuma	+
crp	fumb	+
crp	fumc	+
crp	fur	+
crp	gabd	+
crp	gabp	+
crp	gabt	+
crp	gada	-
crp	gadb	-
crp	gadc	-
crp	gade	-
crp	gadx	-
crp	gale	+
crp	gale	-
crp	galk	+
crp	galk	-
crp	galm	+
crp	galm	-
crp	galp	+
crp	gals	+
crp	galt	+
crp	galt	-
crp	gapa	+

crp	gata	+
crp	gatb	+
crp	gatc	+
crp	gatd	+
crp	gaty	+
crp	gatz	+
crp	gcd	-
crp	gcvh	+
crp	gcvp	+
crp	gcvt	+
crp	gdha	-
crp	glcc	+
crp	glga	+
crp	glgc	+
crp	glgp	+
crp	glgs	+
crp	glna	+
crp	glna	-
crp	glng	+
crp	glng	-
crp	glnl	+
crp	glnl	-
crp	glpa	+
crp	glpb	+
crp	glpc	+
crp	glpd	+
crp	glpe	+
crp	glpf	+
crp	glpg	+
crp	glpk	+
crp	glpq	+
crp	glpr	+
crp	glpt	+
crp	glpx	+
crp	glta	+
crp	gltb	-
crp	gltd	-
crp	gltf	-
crp	gntk	+
crp	gntp	+
crp	gntt	+
crp	gntu	+
crp	gntx	+
crp	gpsa	+
crp	grpe	+
crp	guaa	+
crp	guab	+
crp	gutm	+
crp	gutq	+

crp	gyra	+
crp	hflc	-
crp	hflk	-
crp	hflx	-
crp	hfq	-
crp	hlye	+
crp	hofb	+
crp	hofc	+
crp	hofm	+
crp	hofn	+
crp	hofo	+
crp	hofp	+
crp	hpt	+
crp	hupa	+
crp	hupb	+
crp	hyfa	+
crp	hyfb	+
crp	hyfc	+
crp	hyfd	+
crp	hyfe	+
crp	hyff	+
crp	hyfg	+
crp	hyfh	+
crp	hyfi	+
crp	hyfj	+
crp	hyfr	+
crp	idnd	+
crp	idnk	+
crp	idno	+
crp	idnr	+
crp	idnt	+
crp	ilvb	+
crp	ilvn	+
crp	infb	-
crp	ivbl	+
crp	kbaz	+
crp	laca	+
crp	laca	-
crp	laci	+
crp	lacy	+
crp	lacy	-
crp	lacz	+
crp	lacz	-
crp	lamb	+
crp	lpd	+
crp	lpd	-
crp	lsra	+
crp	lsrb	+
crp	lsrc	+

crp	lsrd	+
crp	lsrf	+
crp	lsrg	+
crp	lsrk	+
crp	lsrr	+
crp	male	+
crp	malf	+
crp	malg	+
crp	mali	+
crp	mali	-
crp	malk	+
crp	malm	+
crp	mals	+
crp	malt	+
crp	malx	+
crp	maly	+
crp	mana	+
crp	mana	-
crp	manx	+
crp	many	+
crp	manz	+
crp	mara	+
crp	marb	+
crp	marr	+
crp	mdh	+
crp	mdte	-
crp	mdtf	-
crp	mela	+
crp	melb	+
crp	melr	+
crp	metk	-
crp	mety	-
crp	mgla	+
crp	mglb	+
crp	mglc	+
crp	mhpa	+
crp	mhpb	+
crp	mhpc	+
crp	mhpd	+
crp	mhpe	+
crp	mhpf	+
crp	mhpr	+
crp	moda	+
crp	modb	+
crp	modc	+
crp	mpl	+
crp	mtla	+
crp	mtld	+
crp	mtlr	+

crp	murp	+
crp	murq	+
crp	nadc	+
crp	nana	+
crp	nanc	+
crp	nane	+
crp	nank	+
crp	nanm	+
crp	nant	+
crp	narq	-
crp	nfua	+
crp	nirb	-
crp	nirc	-
crp	nird	-
crp	nlpe	-
crp	nmpc	+
crp	nrda	+
crp	nrdb	+
crp	nupc	+
crp	nupg	+
crp	nupg	-
crp	nusa	-
crp	ompa	+
crp	ompf	+
crp	ompr	+
crp	ompr	-
crp	ompw	-
crp	osmy	-
crp	oxyr	+
crp	paaa	+
crp	paab	+
crp	paac	+
crp	paad	+
crp	paae	+
crp	paaf	+
crp	paag	+
crp	paah	+
crp	paai	+
crp	paaj	+
crp	paak	+
crp	pck	+
crp	pck	-
crp	pdhr	+
crp	pflb	+
crp	pgk	+
crp	pncb	-
crp	pnp	-
crp	ppdd	+
crp	ppia	-

crp	prib	+
crp	prop	+
crp	prop	-
crp	prpb	+
crp	prpc	+
crp	prpd	+
crp	prpe	+
crp	prpr	+
crp	psie	-
crp	ptsg	+
crp	ptsh	+
crp	ptsh	-
crp	ptsi	+
crp	ptsi	-
crp	putp	-
crp	puub	-
crp	puuc	-
crp	puud	-
crp	puue	-
crp	puur	-
crp	raia	+
crp	rbfa	-
crp	rbsa	+
crp	rbsb	+
crp	rbsc	+
crp	rbsd	+
crp	rbsk	+
crp	rbsr	+
crp	rela	+
crp	rhaa	+
crp	rhab	+
crp	rhad	+
crp	rhar	+
crp	rhas	+
crp	rhat	+
crp	rmf	+
crp	rof	-
crp	rpli	+
crp	rplm	-
crp	rpoh	+
crp	rpoh	-
crp	rpos	+
crp	rpsf	+
crp	rpsi	-
crp	rpso	-
crp	rpsr	+
crp	sbmc	+
crp	sdha	+
crp	sdhb	+

crp	sdhc	+
crp	sdhd	+
crp	secb	+
crp	sera	+
crp	serc	-
crp	sfsa	+
crp	sgbe	+
crp	sgbh	+
crp	sgbu	+
crp	soda	+
crp	sodb	-
crp	sohb	-
crp	spec	-
crp	spf	-
crp	srla	+
crp	srlb	+
crp	srld	+
crp	srle	+
crp	srlr	+
crp	suca	+
crp	sucb	+
crp	succ	+
crp	sucd	+
crp	sxy	+
crp	tam	+
crp	tdca	+
crp	tdcb	+
crp	tdcc	+
crp	tdcd	+
crp	tdce	+
crp	tdcf	+
crp	tdcg	+
crp	tnaa	+
crp	tnab	+
crp	tnac	+
crp	treb	-
crp	trec	-
crp	trg	+
crp	trub	-
crp	trxa	-
crp	ubig	+
crp	udp	+
crp	ugpa	+
crp	ugpb	+
crp	ugpc	+
crp	ugpe	+
crp	ugpq	+
crp	uhpt	+
crp	uida	+

crp	uidb	+
crp	uidc	+
crp	ulaa	+
crp	ulab	+
crp	ulac	+
crp	ulad	+
crp	ulae	+
crp	ulaf	+
crp	uxaa	+
crp	uxab	+
crp	uxac	+
crp	uxua	+
crp	uxub	+
crp	uxur	+
crp	xsea	-
crp	xyla	+
crp	xylb	+
crp	xylf	+
crp	xylg	+
crp	xylh	+
crp	xylr	+
crp	yaep	-
crp	yaeq	-
crp	ybfn	+
crp	ybit	+
crp	ycdz	+
crp	ychh	+
crp	yeip	+
crp	yeiw	+
crp	yfae	+
crp	ygea	+
crp	yhch	+
crp	yhfa	+
crp	yhfa	-
crp	yiaj	-
crp	yiak	+
crp	yial	+
crp	yiam	+
crp	yian	+
crp	yiao	+
crp	yjch	+
crp	yjiy	+
crp	ynfk	+
crp	zrar	+
crp	zras	+
mhpe	mtla	-
mhpe	paaf	-
mhpe	rpmc	-
mhpe	tolc	-

mhpe	ygic	-
mhpe	#N/A	+
mhpe	#N/A	-
mhpe	csga	-
mhpe	galk	-
mhpe	hcp	+
mhpe	hlye	+
mhpe	hpt	+
mhpe	polb	-
mhpe	prok	-
mhpe	psie	-
mhpe	purn	-
mhpe	rpmc	-
mhpe	rpso	-
mhpe	rutg	+
mhpe	sera	+
mhpe	sohb	+
mhpe	srle	+
mhpe	sucb	+
mhpe	tdcf	+
mhpe	#N/A	-
mhpe	#N/A	-
mhpe	#N/A	-
mhpe	#N/A	-
mhpe	#N/A	-
mhpe	#N/A	-
mhpe	cyda	+
mhpe	cydb	+
mhpe	cydc	+
mhpe	cydd	+
mhpe	cyoa	-
mhpe	cyob	-
mhpe	cyoc	-
mhpe	cyod	-
mhpe	cyoe	-
mhpe	dcta	-
mhpe	dcuc	+
mhpe	dpia	+
mhpe	dpib	+
mhpe	fada	-
mhpe	fadb	-
mhpe	fadd	-
mhpe	fade	-
mhpe	fadh	-
mhpe	fadi	-
mhpe	fadj	-
mhpe	fadl	-
mhpe	fnr	-
mhpe	foca	+

mhpe	fuma	-
mhpe	fumb	+
mhpe	fumc	-
mhpe	gada	+
mhpe	gade	+
mhpe	gadx	+
mhpe	gata	-
mhpe	gatb	-
mhpe	gatc	-
mhpe	gatd	-
mhpe	gaty	-
mhpe	gatz	-
mhpe	glca	-
mhpe	glcb	-
mhpe	glcd	-
mhpe	glcf	-
mhpe	glcg	-
mhpe	glpa	-
mhpe	glpb	-
mhpe	glpc	-
mhpe	glpd	-
mhpe	glta	-
mhpe	hema	+
mhpe	hyaa	+
mhpe	hyab	+
mhpe	hyac	+
mhpe	hyad	+
mhpe	hyae	+
mhpe	hyaf	+
mhpe	hyba	-
mhpe	hybb	-
mhpe	hybc	-
mhpe	hybd	-
mhpe	hybe	-
mhpe	hybf	-
mhpe	hybg	-
mhpe	hybo	-
mhpe	icd	-
mhpe	lldd	-
mhpe	lldp	-
mhpe	lldr	-
mhpe	lpd	-
mhpe	mdh	-
mhpe	moea	+
mhpe	moeb	+
mhpe	ndh	-
mhpe	nuoa	-
mhpe	nuob	-
mhpe	nuoc	-

mhpe	nuoe	-
mhpe	nuof	-
mhpe	nuog	-
mhpe	nuoh	-
mhpe	nuoi	-
mhpe	nuoj	-
mhpe	nuok	-
mhpe	nuol	-
mhpe	nuom	-
mhpe	nuon	-
mhpe	ompw	-
mhpe	oppa	-
mhpe	oppb	-
mhpe	oppc	-
mhpe	oppd	-
mhpe	oppf	-
mhpe	pflb	+
mhpe	prfa	+
mhpe	prmc	+
mhpe	ptsg	-
mhpe	puua	-
mhpe	puub	-
mhpe	puuc	-
mhpe	puud	-
mhpe	puue	-
mhpe	puup	-
mhpe	puur	-
mhpe	rhat	-
mhpe	rplb	-
mhpe	rplc	-
mhpe	rpld	-
mhpe	rplp	-
mhpe	rplv	-
mhpe	rplw	-
mhpe	rpmc	-
mhpe	rpos	-
mhpe	rpsc	-
mhpe	rpsj	-
mhpe	rpsq	-
mhpe	rpss	-
mhpe	ruta	+
mhpe	rutb	+
mhpe	rutc	+
mhpe	rutd	+
mhpe	rute	+
mhpe	rutf	+
mhpe	rutg	+
mhpe	sdha	+
mhpe	sdha	-

mhpe	sdhb	+
mhpe	sdhb	-
mhpe	sdhc	+
mhpe	sdhc	-
mhpe	sdhd	+
mhpe	sdhd	-
mhpe	soda	-
mhpe	ssb	-
mhpe	suca	+
mhpe	suca	-
mhpe	sucb	+
mhpe	sucb	-
mhpe	succ	+
mhpe	succ	-
mhpe	sucd	+
mhpe	sucd	-
mhpe	tpx	-
mhpe	treb	+
mhpe	trec	+
mhpe	ubia	-
mhpe	ubic	-
mhpe	uvra	-
mhpe	xylr	+
mhpe	ybdn	+
mhpe	ydea	-
